# Seasonal population dynamics and the genetic structure of the mosquito vector *Aedes aegypti* in São Paulo, Brazil

**DOI:** 10.1002/ece3.392

**Published:** 2012-10-09

**Authors:** Melina Campos, Carine Spenassatto, Maria Lourdes da Graça Macoris, Karina dos Santos Paduan, João Pinto, Paulo Eduardo Martins Ribolla

**Affiliations:** 1 Departamento de Parasitologia, Instituto de BiociênciasUNESP, Botucatu, São Paulo, Brazil; 2 Superintendencia de Controle de Endemias, SUCENMarília, São Paulo, Brazil; 3 UEI Parasitologia Médica/Centro de Malária e outras Doenças Tropicais, Instituto de Higiene e Medicina Tropical, Universidade Nova de LisboaPortugal

**Keywords:** *Aedes aegypti*, population genetics, SNPs, dengue.

## Abstract

Population genetic studies of insect vectors can generate knowledge to improve epidemiological studies focused on the decrease of pathogen transmission. In this study, we used nine SNPs across the *Aedes aegypti* genome to characterize seasonal population variations of this important dengue vector. Mosquito samples were obtained by ovitraps placed over Botucatu SP from 2005 to 2010. Our data show that, regardless of the large variation in mosquito abundance (deduced from the number of eggs obtained from ovitraps), the effective population size remained stable over the years. These results suggest that *Ae. aegypti* is able to maintain a sufficiently large active breeding population during the dry season to keep genetic frequencies stable. These results open new perspectives on mosquito survey and control methods.

## Introduction

**Figure 1:**
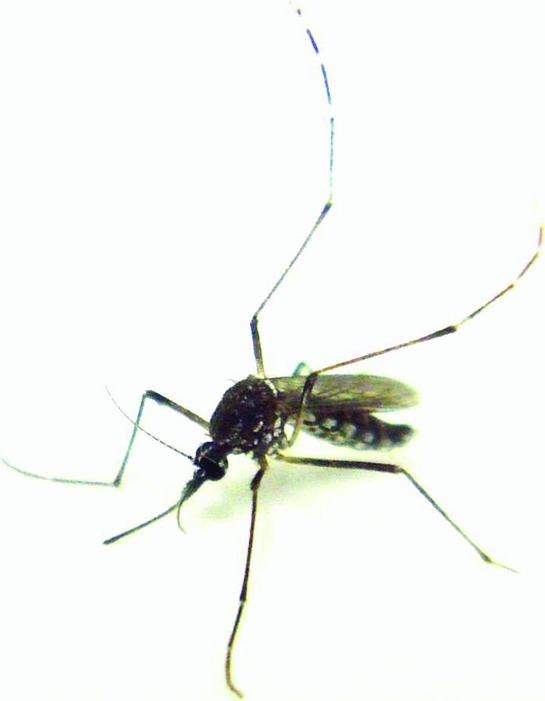
*Aedes aegypti* female mosquito. Photograph with a digital camera SC30 (OLYMPUS) under a stereomicroscope OLYMPUS SZ61 (12× magnification).

The recent history of dengue in Brazil is a remarkable example of the (re-)emergence of a vector-borne disease at a national scale. Its primary vector *Aedes aegypti* L. 1762 (Fig. 1) was reintroduced in the country during the 1970s (Amaral and Tauil 1983; Monath 1994; Gubler and Kuno 1997) and in 1986, there was the introduction of dengue virus in Brazil (Teixeira et al. 2009). Today, Brazil has one of the world's highest annual number of dengue cases (Lindoso and Lindoso 2009).

A major factor contributing for this phenomenon is the highly anthropogenic ecology of *Ae. aegypti* (Kuno 1995). Most common breeding sites explored by this mosquito, such as water tanks, tires, gutters, and plant vases, are intimately associated with human activity. Increases in human population density caused by urbanization, intensive use of artificial containers, and a tropical climate form ideal environmental conditions for *Ae. aegypti* proliferation in Brazil (Labeaud 2008). As a consequence, this urban vector is now widespread throughout Brazil (Schatzmayr 2000; Nogueira et al. 2002).

Despite the fact that many of the breeding sites explored by *Ae. aegpytpi* are of permanent nature, mosquito density and dengue transmission are strongly correlated with climate (Huber et al. 2000; Focks 2003; Vezzani et al. 2004; Dibo et al. 2008; Fávaro et al. 2008). Adult mosquito abundance peaks with high temperature and humidity during the summer and decreases during the cold dry season (Huber et al. 2000). However, little is known about possible genetic variations associated with the seasonal population dynamics of this mosquito. In this context, three scenarios can be hypothesized: (1) a reduction in mosquito density due to the influence of seasonal fluctuation and insecticide treatment has the potential to alter the genetic variability of populations (Lerdthusnee & Chareonviriyaphap 1999); (2) the fact that mosquito eggs are resistant to desiccation for months allowing survival during the cold dry season could suggest a more stable effective population size (Russell et al. 2001); (3) some researches have demonstrated high *Ae. aegypti* dispersal, that could suggest a more dynamic situation characteristic of a metapopulation with annual local extinctions in the dry season followed by re-introduction of migrants during the rainy season (Trpis et al. 1995; Reiter and Gubler 1997).

These three hypotheses lead to different situations regarding mosquito survey and control. A more stable situation could favor the selection of important phenotypes, such as insecticide-resistance lineages. Annual re-introduction of mosquitoes could increase the likelihood of virus introduction.

Several groups are trying to model *Ae. aegypti* population dynamics to propose new control strategies that would decrease dengue transmission (Honório et al. 2009; Aldstadt et al. 2011; Oki et al. 2011). It will be important to include any novel data on population dynamics in such simulations. In this context, the aims of this study were to investigate *Ae. aegypti* population dynamics in a city with marked seasonal climatic variation and to determine associated temporal variations in the genetic diversity of this vector.

## Materials and Methods

### Study sites

**Figure 2:**
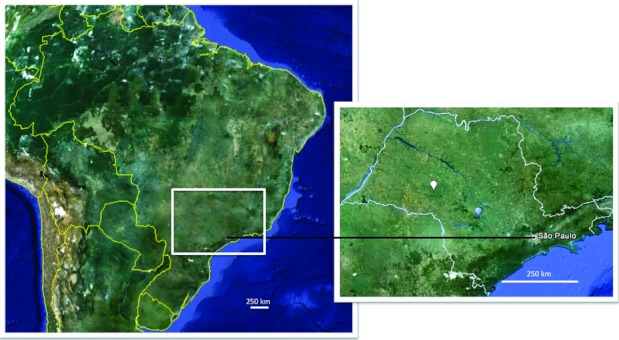
Map of São Paulo state, Brazil, showing the location of Botucatu (blue marker) and Marília (white marker).

**Figure 3:**
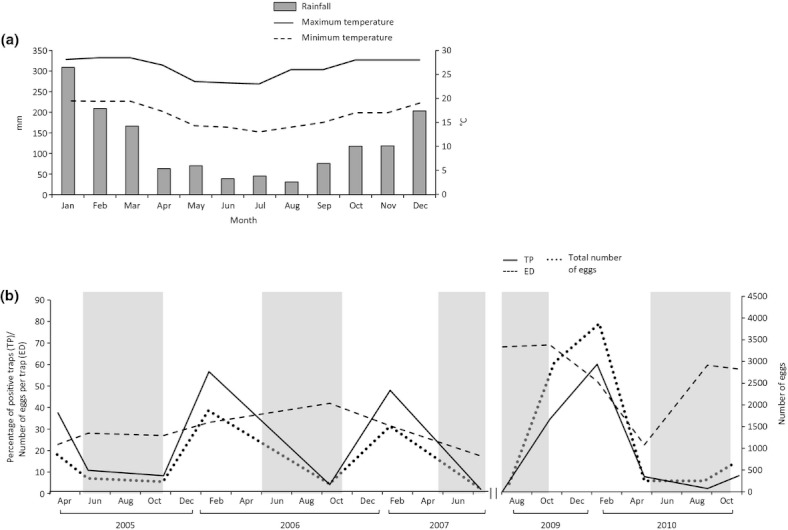
Climate and *Ae. aegypti* entomological indices for Botucatu A) Monthly mean values of maximum/minimum temperature (ºC) and precipitation (mm) for the period of 1995–2007 (data obtained from the Meteorological Institute of Sao Paulo). B) Monthly Estimates of trap positivity index (TP), eggs density index (ED), and total number of eggs for the study period. Shaded areas represent the cold/dry season months.

Botucatu (22º53′S 48º26′W 840 m) is a city located in the midwest of Sao Paulo State, Brazil (Fig. 2, A), with an area of 1,482 km² and 128,788 inhabitants (IBGE 2011). The climate is Humid Middle Latitude; there is a dry season in the winter between May and October, and a hot wet summer between November and April (Fig. 3, A). Marília city (22º12′S 49º56′W 675 m) is also located in Sao Paulo state, *ca*. 200 km northwest of Botucatu and has a similar climatic profile.

### Mosquito collections

Mosquito collections were carried out in Botucatu by the local Epidemiological Surveillance Service using oviposition traps (ovitraps) (Fay and Eliason 1966). Traps consisted of a wooden stick (13 cm × 2.5 cm) in a black plastic container filled with water and an infusion of diluted hay. The distribution of ovitraps covered the entire municipality, according to the number of blocks and dwellings. One-hundred and twenty ovitraps were placed, one per block. Thirteen collections were made from April 2005 to August 2007 and from August 2009 to November 2010. At least two collections were made for each year, one during the rainy season and the other in the dry season. One week after the placement of the ovitraps, wooden sticks were removed and inspected for eggs in the laboratory. Two indices were calculated from ovitrap data: trap positivity index (TP), which is the percentage of positive traps; eggs density index (ED), which is the ratio between the total number of eggs and the number of positive traps (Gomes 1998). Eggs were reared until the adult stage under controlled conditions (27 ± 1°C; 80 ± 5% RH and 15 h light/dark regime) in an insectary. After morphological identification using the key of Consoli and Lourenço-de-Oliveira (1994), adult *Ae. aegypti* were frozen at −20°C until DNA extraction.

Mosquito collections took place in Marília during the rainy season of 2007, also using ovitraps. Mosquitoes were reared to adults and preserved as described above.

### SNP genotyping

DNA was extracted with Chelex100® Molecular Biology Grade resin (Bio-Rad Laboratories), prepared at 5%. DNA concentration was measured on a NanoDrop® spectrophotometer (ND-1000) and samples were stored at −20°C.

Nine Single Nucleotide Polymorphims were genotyped. Of these, seven SNP markers were described in Paduan and Ribolla (2009) and two additional ones were tested for this study. The nine SNPs are located at the following genes: AeIMUC1 (Mucin-like protein), apolLp-II (Apolipophorin II), Ef-2 (Elongation factor), Na/K (Sodium/potassium channel), PGK (Phosphoglycerate kinase), CYP9J2 (Cytocrome P450), Chym (Chymotripsin), Ferho (Ferritin), and TSF (Transferrin). These genes are located in different regions of the three chromosomes of this mosquito, which minimizes the effect of linkage disequilibrium (Severson et al. 2002; Paduan and Ribolla 2009). TaqMan® (Minor Groove Binder – MGB) primers and probes (Assay-by-design 40x) were constructed for these SNP markers by Applied Biosystems. Probes were labeled with two different allele-specific fluorophores, VIC™ and FAM™. Samples were organized in 96-well plates and four wells per plate were occupied by three positive controls and one negative control. Positive controls consisted of known genotypes, one homozygote for each allele and one heterozygote. Negative controls contained all reagents, but no DNA template. PCR reactions were prepared in a Corbett Robotics robot (Corbett Life Science) using the reagent kit 2× QuantiFast Probe PCR Master Mix (Qiagen) as described by the manufactor. The DNA of each individual mosquito was amplified by real-time PCR using the platform StepOnePlus™ v. 2.1 (Applied Biosystems) and optical system for TaqMan® allelic discrimination. Initial conditions for amplification were 3 min at 95°C (Taq DNA polymerase activation) followed by 40 cycles at 92°C for 30 sec (denaturation), and 60°C for 30 sec (annealing/extension). Determination of alleles was calculated by a ratio between fluorescence of the fluorophores FAM™ and VIC™.

### Data analysis

Observed heterozygote frequencies were tested against Hardy–Weinberg proportions by exact tests (Guo and Thompson 1992) available in ARLEQUIN 3.0 software (Excoffier et al. 2005). Analysis of molecular variance (AMOVA) was used to assess population structure (Excoffier et al. 1992). The fixation index (*F*_*ST*_), calculated according to Weir and Cockerham (1984), was used as an estimator of genetic differentiation between groups. Pairwise *F*_*ST*_ estimates were calculated between sample pairs. Significance of *F*_*ST*_ values was verified by nonparametric permutation tests also available in ARLEQUIN (Excoffier et al. 2005).

STRUCTURE 2.3 (Pritchard et al. 2000) was used to determine population structure based on multilocus genotypic data. Cluster analysis was performed with prior information about collection year and with the option of admixture*,* that is*,* assuming a degree of ancestry within individuals. Ten replicate independent runs, each with 50,000 iterations after a burn-in length of 10,000 iterations, were performed for the ΔK statistic (Evanno et al. 2005) was used to infer the best number of clusters (K).

Estimates of effective population size (Ne) were calculated from genetic data using the linkage disequilibrium (LD) method described by Hill (1981) and implemented in the software NeESTIMATOR (Ovenden et al. 2007). In order to avoid possible bias due to seasonality, Ne estimates were based only in samples collected during the rainy season of each year.

## Results

### Ovitrap data

Fluctuations in total egg abundance and TP followed a pattern associated with precipitation and temperature (Fig. 3, B). During the dry season, the mean of monthly values of total egg abundance and TP were 204 and 5.5%, respectively. In the rainy season, these values increased to 1710 eggs and 38.1% positive ovitraps. A similar seasonal pattern was not observed for ED estimates (Fig. 3, B). These remained relatively stable between April 2005 and August 2007, varying between 18 eggs/trap and 42 eggs/trap. In the period between August 2009 and November 2010, there was a decrease in ED during the last months (February–April) of the rainy season until, but these values started to increase after May, at the beginning of the dry season. The values of ED did not correlate with the estimates of TP (Spearman's *r* = 0.047, *P* = 0.879).

### Genetic data

**Figure 4:**
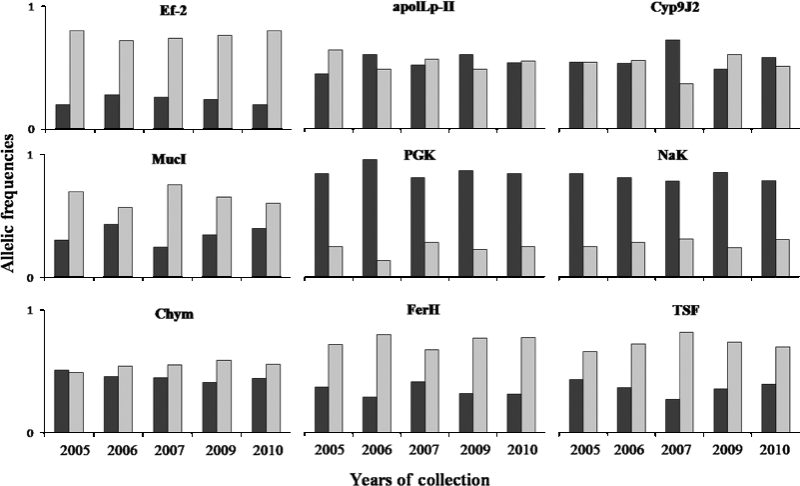
Allele frequencies for the nine SNP markers genotyped. Vertical bars represent the relative frequency of each allele (di-allelic loci), by year of collection.

Forty-six individuals from 2005, 2006, 2007, and 2009 collections, and ninety-two individuals from 2010 were genotyped. Analyses were performed by grouping mosquito samples annually. Allelic frequencies at each SNP remained relatively stable over the years (Fig. 4). Alternation in the most frequent allele between years was observed in three of the nine markers. For these, the relative allele frequency was around 0.5. SNPs with rare allele frequencies (*i.e.,* <10%, Ef-2, NaK, and PGK) did not tend to fixation. The nine SNPs analyzed showed no deviation in genotype frequencies from H-W expectations.

AMOVA results revealed low and nonsignificant fixation indices between samples from Botucatu (*F*_*ST*_ = 0.003, *P* = 0.763). Pairwise *F*_*ST*_ (Table 1) showed one significant result (*F*_*ST*_ = 0.017, *P* < 0.01), between the 2006 and 2007 collections, in comparisons involving temporal samples from Botucatu. In contrast, all Botucatu samples were significantly differentiated from the sample of Marília, with *F*_*ST*_ varying from 0.018 to 0.058 (*P* < 0.01 for all *F*_*ST*_ values).

**Table 1:** Analysis between pairs of years of collections and *out-group*

	2005	2006	2007	2009	2010
2005	–				
2006	0.00584	–			
2007	0.00672	0.01716*	–		
2009	−0.00131	−0.00411	0.00956	–	
2010	−0.00162	−0.00167	0.00886	−0.00360	–
*Marília*	0.01728*	0.02104*	0.05842*	0.02811*	0.02228*

* *P* < 0.01.

**Figure 5:**
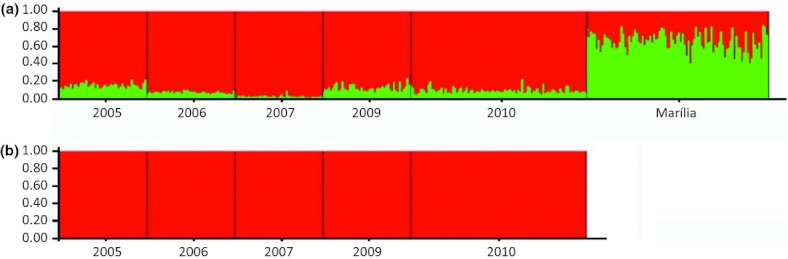
Bayesian clustering analysis (STRUCTURE) conducted with *Ae. aegypti* samples from Botucatu and MaríliaEach individual multilocus genotype is represented by a column partitioned into two colors according to the probability of ancestry to each cluster (K = 2). In the *X*-axis, individuals are ordered according to collection year (for Botucatu) and locality. *Y*-axis: probability of ancestry. A) only temporal sample from Botucatu; B) all samples.

Bayesian clustering analysis implemented by STRUCTURE gave K = 2 as the best cluster number for the whole dataset (Fig 5, A). This partitioning corresponded to the separation of the sample of Marília from the samples of Botucatu (Fig. 5). All temporal samples from Botucatu were grouped in the same genetic cluster. This clustering was confirmed when analysis was repeated excluding the sample from Marília. In this case, the best number of clusters was K = 1 (Fig. 5, B).

**Figure 6:**
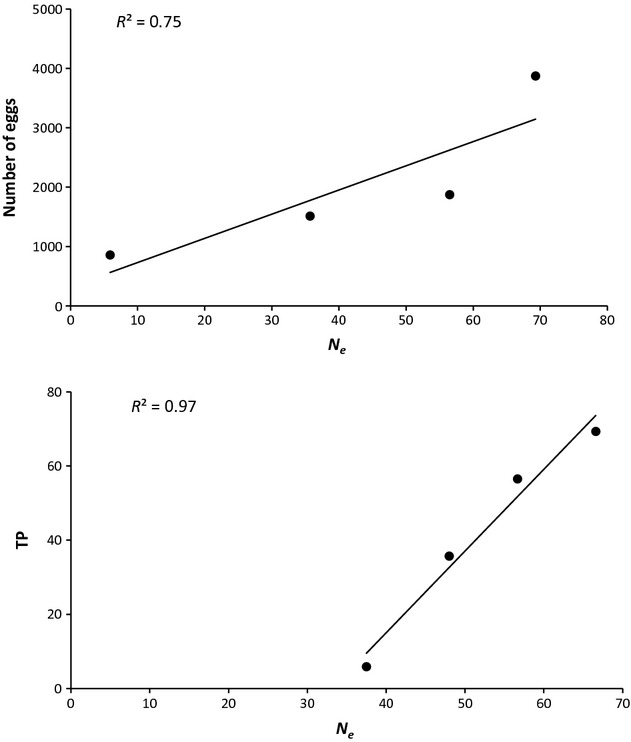
Scatter-plots of Ne estimates against total number of eggs (A) and trap positivity (B).

Estimates of effective population size at the rainy season per year of collection are shown in Table 2. Values ranged from 152 to infinity between 2007 and 2010, with overlapping confidence intervals ranging from 36 to infinity. The sample of 2005 showed a significantly lower Ne estimate, judging by the nonoverlapping confidence interval. In order to compare effective population size with ovitrap indices, we used the lower CI limit value of each Ne estimate for each year to perform a correlation analysis with the maximum monthly value of TP, ED, and total egg number for that year (Fig. 6). Results showed positive correlations between Ne and both TP (*r*^2^ = 0.97) and total number of eggs (*r*^2^ = 0.75).

**Table 2:** Population Size by Linkage disequilibrium estimate of each collection peak by year

Year of Collection	Linkage disequilibrium Estimate	Approx. 95% Confidence Interval
2005	10.2	[5.9–21.3]
2006	∞	[56.5–∞]
2007	152.3	[35.7–∞]
2010	∞	[69.3–∞]

## Discussion

Ovitraps are an effective alternative method for monitoring *Ae. aegypti* abundance (Service 1992), being considered more sensitive than other methods such as larvae collections (Focks 2003; Morato et al. 2005; Dibo et al. 2008). By collecting eggs with ovitraps, we have indirectly analyzed the abundance of adult females in the environment. Results point to a marked seasonality of *Ae. aegypti* in the study area, with abundance reaching a peak during the rainy/warm season and then decreasing during the dry/cold season. This result agrees with previous observations for this species (Huber et al. 2000, 2002; Vezzani et al. 2004; Dibo et al. 2008). A positive relationship between precipitation, humidity and temperature, and dengue vector abundance seems to be obvious. Seasonality affects the mosquito reproductive pattern because precipitation results directly in water level increases in breeding sites (Moore et al. 1978), higher temperatures accelerate larval development time (Tun-Lin et al. 2000), and humidity increases adult fitness (Hales et al. 2002). Nevertheless, each one of these variables may have a different impact on *Ae. aegypti* depending on the local ecology of the vector population (Azil et al. 2010).

Of the three variables estimated from ovitrap data, ED did not follow the same seasonal variation as the other two. In fact, there was a tendency for a decrease in ED values during the rainy season of 2009/2010 as opposed to the pattern of TP and total number of eggs for the same period. While the proportion of positive traps (TP) and total number of eggs are directly influenced by the abundance of adult gravid females, which increases during the rainy/warm season, the same may not hold for ED estimates. The decline in the mean number of eggs per ovitrap (ED) observed during the rainy season may be a consequence of a higher availability of breeding sites alternative to ovitraps. The impact of greater breeding site availability is likely to be lower for the proportion of positive ovitraps (TP) if mosquito densities are high, especially when taking into consideration that *Ae. aegypti* gravid females tend to disperse their eggs over more than one breeding site (Apostol et al. 1994). Conversely, during the dry season, a smaller but still actively breeding mosquito population would tend to concentrate eggs in the fewer breeding sites available, such as ovitraps that are closest to resting sites.

The presence of an active breeding population of *Ae. aegypti* during the cold/dry season, as evidenced by the presence of eggs in ovitraps, agrees with an apparent genetic stability observed by genotyping of SNP markers. From the seasonal fluctuations in mosquito abundance recorded, one might expect expansion of residual local population and/or new colonization by migrants at the beginning of each rainy season (Huber et al. 2002). The relative stability in allele frequencies found over a period of 5 years does not seem to support the hypothesis of dramatic seasonal changes in effective population size. Such changes could have impacted levels of genetic differentiation between temporal samples, which was not observed by Bayesian Clustering analysis or by pairwise *F*_*ST*_ estimates. Furthermore, introduction of migrants could have an important role in modifying allelic composition. This was observed by Paduan and Ribolla (2008) in *Ae. aegypti* populations from Santos, the major port city in Brazil. This study detected several mtDNA COI haplotypes in Santos that were absent in samples from other Brazilian cities. Estimates of Ne were also comparable between years and the positive correlation between these and ovitrap indices (as predictors of abundance) may indicate a relatively high reproductive success of *Ae. aegypti* in the study area. Altogether, these results suggest that in Botucatu, *Ae. aegypti* is able to maintain an active breeding population during the dry season with an effective population size sufficiently large to sustain genetic variability over time. Furthermore, the capacity of *Ae. aegypti* eggs in resisting to desiccation over the dry season until hatching with the onset of the first rains can also contribute for the maintenance of a relatively stable effective population size.

A limitation of the genetic analysis conducted in this study was the low number of SNP markers genotyped, which could have influenced some of the statistics used. The nine SNPs were, however, sufficient to detect significant genetic differentiation between Botucatu and Marília samples, located *ca*. 200 km apart. The *F*_*ST*_ estimates between the two localities (0.017–0.058) were comparable to those obtained in previous studies on the genetic structure of *Ae. aegypti* based on more polymorphic markers such as microsatellites and involving samples collected at similar geographic distances (Huber et al. 2004; Endersby et al. 2009). However, this does not preclude a possible lack of sensitivity to detect lower levels of differentiation that may occur between temporal samples collected from the same sampling area. The analysis of a larger number of SNPs or the inclusion of more polymorphic genetic markers (*e.g.,* microsatellites) would be required to confirm these results.

The observations made in this study argue for the implementation of vector control measures targeting immature stages of *Ae. aegypti* during the dry season. Although eliminating breeding sites is a virtually impossible task during the rainy season, concentration of oviposition into fewer breeding sites during the dry season might increase the feasibility of such a control strategy. This would involve geo-referencing of the most productive oviposition sites of the area and the application of efficient trapping methods such as sticky ovitraps, in which the ovipositing female is also caught. Such an approach could result in a significant reduction in *Ae. aegypti* effective population size during the dry season, which might in turn affect mosquito abundance during the rainy season and consequently dengue transmission.
